# Exploring Out-of-Pocket Costs and Health-Related Quality of Life among Patients with Cancer and Diabetes in Malaysia

**DOI:** 10.21315/mjms-07-2025-562

**Published:** 2025-12-31

**Authors:** Mohd Redhuan Dzulkipli, Asrul Akmal Shafie, Siti Noorsuriani Maon, Azuana Ramli, Abdul Haniff Mohammad Yahaya, Ho See Wan, Nor Ilham Ainaa Muhsin, Azmi Nor Mohd Farez Ahmat

**Affiliations:** 1 Discipline of Social and Administrative Pharmacy, School of Pharmaceutical Sciences, Universiti Sains Malaysia, Pulau Pinang, Malaysia; 2 Faculty of Business and Management, Universiti Teknologi MARA Selangor, Puncak Alam Campus, Selangor, Malaysia; 3 Centre of Compliance and Quality Control, National Pharmaceutical Regulatory Agency, Ministry of Health Malaysia, Petaling Jaya, Selangor, Malaysia; 4 Pharmacy Policy and Strategic Planning Division, Ministry of Health Malaysia, Petaling Jaya, Selangor, Malaysia; 5 Pharmacy Practice and Development Division, Ministry of Health Malaysia, Putrajaya, Malaysia

**Keywords:** out-of-pocket costs, health-related quality of life, financial hardship

## Abstract

**Background:**

Out-of-pocket costs for chronic illnesses have become significant barriers to continuing treatment, given the already substantial cost of the diseases. This study aims to estimate out-of-pocket costs and determine the health-related quality of life of patients with cancer and diabetes.

**Methods:**

A purposive cross-sectional study (January 2021–January 2022) was conducted to assess health-related quality of life and out-of-pocket costs among Malaysian adults with cancer or diabetes. Using the validated EQ-5D-5L and a three-section questionnaire, 571 participants from three hospitals and online surveys provided data on five cost categories expressed in 2021 MYR and USD.

**Results:**

Significant differences were observed across all cost categories between patients with cancer and diabetes. Diagnostic imaging had a larger effect size (*P* < 0.0001, *r* = 0.467). Annual outpatient and hospitalisation costs among patients with cancer were found to be 42 and 11 times higher than for patients with diabetes, respectively. Overall, the out-of-pocket expenditure per year for patients with cancer was MYR 88,346.12 and MYR 9,010.60 for patients with diabetes. Additionally, patients with cancer rated their health-related quality of life using the EQ-VAS score lower than patients with diabetes (EQ-VAS = 63.7, SD = 19.2 and EQ-VAS = 71.6, SD = 18.7).

**Conclusion:**

Cancer and diabetes impose significant financial and quality of life burdens on patients in Malaysia. This study highlights gaps in insurance coverage and public assistance, calling for more comprehensive support for medical and nonmedical expenses and psychological support for patients. The analysis, which focuses on annual costs, may underestimate the long-term financial impact, especially for patients with cancer. Future research should examine household spending and the ability to pay for treatment costs to better understand the full economic burden of these chronic diseases.

## Introduction

Cancer is increasingly becoming a significant contributor to mortality in Malaysia. As the country advances toward becoming a developed nation, it is likely to witness a substantial rise in cancer cases reported within its healthcare system. This growing prevalence of cancer may lead to several associated challenges, including a financial burden on patients and potential barriers to accessing necessary treatments. Patients with cancer who have lost the ability to perform daily living, live alone, have an income lower than average, or lack health insurance were found to experience higher out-of-pocket costs (OOPs) (1). This may delay treatment and incur additional costs to the healthcare system as their disease progresses, necessitating the need for intensive medical intervention (2). To a certain extent, the cost of medical care has forced patients, especially in Malaysia, to sell their assets and resort to borrowing to fund the cost of illnesses (3). Some patients with cancer may choose to forgo treatment and prioritise their financial situation due to the dilemma of either undergoing cancer treatment versus managing daily living expenses. Although medical treatment and chemotherapy drugs are available at subsidised rates through selected public hospitals, insurance may not cover out-of-pocket costs for services such as transportation, outpatient care, imaging, and laboratory tests at private hospitals or clinics. This leads to a significant financial burden on healthcare expenditure for patients with cancer (4, 5). As for diabetes mellitus (DM), it has been reported that the disease has cost the nation almost 13% of the annual national budget or MYR 2 billion per year (6). A World Bank report has noted that the rise of DM and cancer among younger populations may cause growing concern (7). These diseases are associated with high treatment costs that could lead to financial distress (7, 8). In another study, the diabetes cost in Malaysia was estimated to be at USD 600 million (9). This raises legitimate concerns about the increasing cost of the national budget.

Despite the growing financial burden and reduced quality of life, there was a lack of comparative evidence on the costs and health-related quality of life (HRQoL) among patients with cancer and DM, especially in Malaysia, is lacking. Therefore, an in-depth comparison can help clarify the burden associated with each of the chronic diseases experienced by patients and allow for better prioritisation of resource allocation, thus paving the way for more funding to improve medicines and personalised insurance policy premiums for each disease. This study compared out-of-pocket costs and HRQoL between patients with cancer and DM in Malaysia using the EQ-5D-5L and EQ-VAS instruments. It also identifies the key domains of impairment and financial burden among patients with cancer and DM.

## Methods

### Study Design, Population and Sample Size

A purposive, cross-sectional study was conducted from January 2021 to January 2022 to determine HRQoL and out-of-pocket costs in Malaysia. The target population of this study comprised adult Malaysian patients aged 18 years and above who had been diagnosed with either cancer or DM. Eligibility for cancer patients included patients undergoing treatment and those who have completed treatment, regardless of the cancer type or stage. Inclusion for the comparative group was limited to patients with type 2 DM, a highly prevalent and manageable chronic condition in Malaysia. Type 2 DM was selected to compare the economic and quality of life impacts of a non-communicable, controllable disease with those of a more severe and invasive illness, such as cancer (10–13).

Initially, the study’s data collection was intended to take place in three selected public hospitals in the Klang Valley. The participating hospitals are Institut Kanser Negara at Putrajaya, Hospital Tengku Ampuan Rahimah at Klang, and Hospital Umum Sarawak. However, due to restrictions imposed during the COVID-19 pandemic in mid-2021, the data collection strategy was adapted to include an online survey administered at that time. To increase the number of potential participants, an alternative online data collection approach using Microsoft Forms was employed. The site investigators received training on data collection in the wards and at a cancer outpatient clinic.

Additionally, an advertisement featuring a survey link and QR code was posted on the notice board of the participating hospital. A total of 674 eligible samples were collected. However, 103 samples were excluded because the respondents were neither patients with DM nor those with cancer, as outlined in the inclusion criteria. This resulted in a final sample size of 571. The final sample sizes were deemed sufficient and within the recommended range, as outlined in the literature (14–16). The costs were valued in 2021 Malaysian Ringgit (MYR) and converted to 2021 US Dollars. The conversion rate was retrieved from the Malaysia Central Bank (USD 1 = MYR 4.18) (31 December 2021, 11:30 AM).

### Instrument

The study employed a three-section survey instrument to gauge respondents’ sociodemographic profiles, HRQoL, and out-of-pocket costs associated with seeking treatment. The EQ-5D-5L, a standard measurement developed by EuroQol, was used to measure the HRQoL (17, 18). The five cost categories querying the patient’s out-of-pocket costs were as follows: outpatient costs, which include expenses associated with outpatient visits, hospitalisation costs incurred during inpatient admissions; laboratory and medical imaging costs that need to be paid out-of-pocket; and transportation costs incurred when travelling to seek cancer treatment. Experts in the relevant field thoroughly tested and vetted the survey instrument.

### Ethical Approval

Ethical approval for the study was obtained from the National Medical Research Register (NMRR) and the facilities of the Ministry of Health Malaysia, with ID: NMRR-20-2816-56539 (IIR).

### Data Analysis

Frequencies and mean score analyses were employed to describe the demographic profiles of the respondents and HRQoL using EQ-5D-5L. The cost data were not normally distributed; thus, the Mann–Whitney U test was used. The Mann–Whitney U test was performed to investigate the differences between patients with cancer and DM in the five cost categories. To further estimate the respondent’s out-of-pocket costs, a generalised linear method (GLM) analysis was employed to gauge the patient’s out-of-pocket costs. GLM provides a wider expansion of analysis to include more response types, such as binary and count data (19–21). Due to the large number of zeros in the data, negative binomial regression was used to better estimate the relevant cost of both patients with cancer and DM. Each cost was extrapolated to calculate annual costs. Stata Statistical Software: Release 14.2 (Stata Corp LP, College Station, TX, USA) was used for data analysis.

## Results

### Demographic

Table 1 presents the demographic profiles of respondents with DM and cancer. Most of the respondents were female, with 56.4% of the total DM patients being female, with a mean age of 51.9 years old (SD = 19.9) and 61.3% of the total cancer patients being female, with a mean age of 52.2 years old (SD = 15.5). Malay ethnicity was the most common among the respondents for both DM and cancer patients, accounting for 72.8% and 54.8% of the participants, respectively. This distribution aligns with the current population census conducted by the Department of Statistics, Malaysia (DOSM) (22). Most respondents were married, with 65.3% of the DM patients and 69.8% of the cancer patients being married. Additionally, 45.5% of the patients with DM and 50.0% of the patients with cancer had at least a primary or secondary education level.

Regarding employment status, most respondents were employed among patients with DM and cancer, with 57.6% and 59.3% reporting employment, respectively. Notably, 94.6% of patients with DM and 85.2% of patients with cancer were employed in full-time positions. Many DM respondents were government employees (47.3%), whereas approximately 52.5% of the participants in this group worked in the private sector. Both patients with DM and cancer reported having households of 3 to 4 people. Additionally, most respondents in both groups reported monthly household incomes of no more than MYR 5,000. The average monthly household income for the population, as reported by the Department of Statistics, Malaysia, was MYR 5,209 (23).

### Health-related Quality of Life using EQ-5D-5L

As shown in Table 2, approximately 42.4% of the respondents reported having mobility problems, ranging from slight problems to extreme difficulties. Regarding self-care, approximately 27.8% of respondents reported difficulty managing themselves, and most (72.2%) reported being able to look after themselves. Regarding usual activities, 45% of the respondents reported slight to extreme problems in performing their normal activities. More than 64% of the respondents reported pain/discomfort in their daily lives, which is consistent with the majority of the respondents living with chronic illnesses such as DM or cancer. As for the anxiety/depression dimension that associated with the disease, the result showed that most respondents (57.6%) reported slight to extreme anxiety/depression.

Table 2 shows the mean EQ-VAS scores for both DM and cancer patients. The mean score for DM respondents was 71.62 (SD = 18.73), whereas the mean score for cancer respondents was 63.72 (SD = 19.23). The results indicate that the cancer respondents had lower EQ-VAS mean scores than those with DM.

### Out-of-pocket Costs

**Figure 1:**
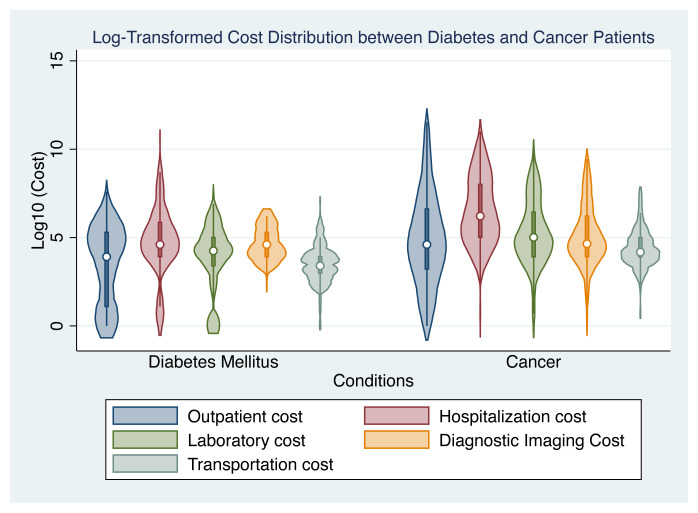
Cost distribution among diabetes and cancer patients

To accurately investigate the cost of having cancer, the researchers studied the out-of-pocket expenditures among patients with cancer and DM. For comparison purposes, the five cost categories (outpatient, hospitalisation, laboratory, diagnostic imaging, and transportation costs) were compared with those of the lesser complex chronic condition DM. Based on the results in Table 3, a significant difference was found between cancer and DM patients across the five cost categories. All cost categories showed statistically significant differences (*P* < 0.05), indicating different cost distributions differ between patients with cancer and DM. The effect sizes for all cost categories ranged from small to large, with diagnostic imaging showing the largest effect between the groups (*P* < 0.0001, *r* = 0.467). The findings were consistent with the visualisation of the violin plot in Figure 1.

The five cost categories were estimated using GLM and negative binomial regression. From the results, the out-of-pocket cost per year for patients with cancer was estimated to be 11 times higher than that for patients with DM (Table 4). Outpatient and hospitalisation costs for patients with cancer were found to be significantly higher than those for patients with DM. The outpatient cost was 42 times higher for cancer patients than for DM patients, with an average of MYR 4,922.83 (SE = 2,249.08) and MYR 116.75 (SE = 17.81) for cancer and DM patients, respectively. The frequency of outpatient visits for cancer patients was five visits per quarter (M = 5.6, SD = 10.9) compared with two visits per quarter (M = 2.1, SD = 1.9) for DM patients quarterly (Table 4). This is equivalent to approximately 10 and 8 annual visits for patients with cancer and DM, respectively. The average cost of hospitalisation was MYR 12,369.69 (SE = 6,236.09), which is 11 times higher than that for patients with DM at MYR 1,110.01 (SE = 604.98). The frequency of hospitalisation was two visits (M = 2.0, SD = 3.4) and at least one visit (M = 0.7, SD = 1.8) quarterly for cancer and DM. Overall, patients with cancer were hospitalised more frequently, approximately eight times annually, compared to two times annually for patients with DM.

## Discussion

### Health-related Quality of Life of the Respondents

The findings revealed differences among the respondents in the HRQoL and perceived health scores, as measured by the EQ-5D-5L. Notably, patients with cancer were more significantly impacted by their condition than those with DM. All the dimensions in EQ-5D-5L were reported to be significantly worse for patients with cancer compared to patients with DM. The finding is consistent with a study among Indian patients with breast cancer, which found a lower utility score compared with non-cancer patients (22). Significant differences were noted for the rest of the EQ-5D-5L dimensions (self-care, usual activities, pain and discomfort, and anxiety and depression) between patients with DM and those with cancer. For example, the self-care dimension showed an increase of 18.7% in the number of cancer patients unable to care for themselves. Approximately 65.7% of the cancer patients reported slight to extreme difficulty continuing their usual activities when they are having cancer, as compared to just 40% of the DM patients reporting having the same problem’s manifestation. Furthermore, pain and discomfort were obviously more severe for the cancer patients than for DM patients, in which an increment of 27% more cancer respondents reported pain and discomfort compared to only 59% of DM patients, which is similar to the finding among the Malaysian cancer population (24). The last dimension of EQ-5D-5L, anxiety and depression, proved that patients with cancer suffered the most compared to those with DM, which is a less chronic disease. Approximately 77% of the cancer respondents reported having anxiety and depression due to having cancer, compared to DM respondents, who reported that 46.1% of the respondents are having the same problem. The findings above are consistent with the study conducted by Malaysian researchers on the health status based on the EQ-5D-5L, which reported pain and discomfort and anxiety and depression dimensions as among the dimensions that were rated at a higher level (24). However, the EQ-VAS score for this study was found to be much lower, which is 63.7 (SD = 19.2), compared to the same cancer patients in a survey conducted by Wan Puteh et al. (25) who scored as high as 81.06 (SD = 16.36).

A notable disparity exists in the HRQoL of patients with DM and those diagnosed with cancer, as evidenced by the study. The mean score of the cancer patients’ EQ-VAS was lower, at 63.7 (SD = 19.2), with a median of, compared to the DM patients’ score of 71.6 (SD = 18.7). Cancer patients experienced lower HRQoL, which might be attributed to the more severe long-term effects of cancer treatments. Many cancer studies have reported the same results over the years (26–28). A study of post-COVID effects among cancer survivors in the United Kingdom found that their anxiety levels have increased, and having less support from their cancer network during the pandemic has affected them and lowered their HRQoL (29). Cancer was undoubtedly perceived as a more severe and chronic condition than other diseases, resulting in lower HRQoL among its patients (30). Cancer is a known disease that may further deteriorate patients’ physical and cognitive function, which contributes to lower HRQoL among patients with cancer (31). These findings reinforced the need for more focused and targeted assistance, such as counselling and moral support, due to the substantial burden borne especially among patients with cancer, thus mitigating the patients’ HRQoL from deteriorating.

### Out-of-pocket Costs among Patients with Cancer

The findings reveal notable differences in healthcare expenses between patients with DM and those with cancer across all categories studied. Negative *Z*-values suggest that individuals with cancer typically face higher costs than patients with DM. The effect sizes ranged from small to large, with the largest difference observed in diagnostic imaging costs (*P* < 0.0001, *r* = 0.467), reflecting a meaningful disparity in the distribution of costs between the two groups. Median values offer a glimpse into typical patient expenses, generally showing higher costs among patients with cancer. The annual cost of cancer screening in the United States supports this finding, which noted that the facility cost in which the testing occurred contributed to the significant cost driver (32). Meanwhile, the interquartile range (IQR) highlights fluctuations in spending, where broader IQRs point to larger variability in costs, which is supported by Greco, Luta, and Wilcox, who emphasised the IQR robustness in skewed and non-normal data distribution (33). The significant effect sizes and Mann–Whitney U test results underscore the substantial differences in cost profiles between the two groups, highlighting the financial burden associated with cancer care.

As for the out-of-pocket expenditure, cancer patient OOP expenditure is 11 times more than for DM patients, as shown in Table 4 in the previous section. All the costs, such as outpatient, hospitalisation, laboratory, diagnostic imaging, and transportation costs, were found to be higher among patients with cancer than among patients with DM. This finding was consistent with the findings from other studies on the out-of-pocket costs among patients with cancer (34). Hospitalisation was the highest cost that contributed to the OOP cost that had to be paid by the patients. The hospitalisation cost of cancer patients was found to be much higher than that of DM patients. For patients with cancer, the cost was an average of MYR 12,369.69 per quarter, which is 11 times higher than that for patients with DM at MYR 1,110.01. This finding was consistent with the findings among oral cancer patients, which recorded the inpatient stay of the patient, especially among early- and late-stage cancer patients, as one of the highest costs that the provider needed to bear in the Malaysian public hospitals (35). The costs for hospitalised patients with cancer were higher, especially in the palliative care phase and for those who lived far from the hospital (36). The farther the house is located, the more the financial burden of financing cancer care among patients may increase (37). In addition, the finding was found to be higher compared to the recent cancer patients in the lower-income group in the Malaysian public healthcare study (38). This could be due to the inclusion of outpatient, hospitalisation, diagnostic imaging, laboratory, and transportation costs as factors.

This study reveals that patients with cancer face significantly poorer HRQoL and far higher out-of-pocket expenditures than patients with diabetes. These disparities call for policies that strengthen financial protection by expanding insurance coverage to include diagnostic imaging, implementing targeted subsidies such as transportation discounts for patients with cancer, and strengthening social safety nets. Overall, cancer care requires a more intensive policy response to improve patient well-being and prevent catastrophic financial hardship.

## Conclusion

Cancer and DM pose significant impacts on patients, particularly on the financial burden and reduced quality of life. In Malaysia’s heavily subsidised healthcare system, it is crucial to examine the entire cost chain to understand the true expenses faced by patients with cancer, particularly the poor and underserved. The findings of this study highlight gaps in the healthcare system resulting from high out-of-pocket costs for patients with cancer and the costs incurred from these chronic diseases, emphasising the need for more comprehensive insurance policy coverage and benefits. This disparity has severe impacts on mental health, daily functioning, and financial stability. Systematic and targeted public assistance should be directed from existing government schemes to address potential shortfalls in cancer and DM care costs.

Additionally, the study may recommend enhanced coverage of both medical and nonmedical expenses to ensure equitable access to healthcare, thereby reducing the financial burden on patients and their families. However, this study has some limitations. The difficulty respondents may have in recalling past events can lead to under- or over-reporting of costs due to recall bias. Consequently, researchers may overlook the long-term economic impact because the costs explored are only for the annual cost rather than the entire treatment duration. This may affect the household’s ability to sustain, especially for long-term cancer treatment. The absence of longitudinal data also precluded the analysis of DM and cancer cost trends. Future studies should investigate the long-term financial impact of both diseases across the full treatment duration rather than annually. Accessing long-term health cost databases is also recommended to support sustainable, viable financing and policy planning.

## Figures and Tables

**Table 1:** Demographic profiles of the respondents (*N* = 571)

Characteristics	Diabetes (*n* = 323)	Cancer (*n* = 248)
Age, mean (SD)	51.9 (14.9)	52.2 (15.5)

Gender, *n* (%)		
Male	141 (43.6)	96 (38.7)
Female	182 (56.4)	152 (61.3)

Ethnicity, *n* (%)		
Malay	235 (72.8)	136 (54.8)
Chinese	46 (14.2)	67 (27.0)
Indian	34 (10.5)	26 (10.5)
Native of Sabah/Sarawak	8 (2.5)	16 (6.5)
Others	-	3 (1.2)

Marital Status, *n* (%)		
Divorced	61 (18.9)	32 (12.9)
Single	51 (15.8)	43 (17.3)
Married	211 (65.3)	173 (69.8)

Education, *n* (%)		
Primary	60 (18.6)	36 (14.5)
Secondary education	147 (45.5)	124 (50.0)
Tertiary education	116 (35.9)	88 (35.5)

Employment status, *n* (%)		
Yes	186 (57.6)	101 (59.3)
No	137 (42.4)	147 (40.7)

Type of employment, *n* (%)		
Part-time employment	10 (5.4)	15 (14.8)
Full-time employment	176 (94.6)	86 (85.2)

Employment sector, *n* (%)		
Government	88 (47.3)	29 (28.7)
Private	78 (41.9)	53 (52.5)
Self-employed	20 (10.8)	19 (18.8)

Household size, *n* (%)		
1 to 2 people	55 (17.0)	44 (17.7)
3 to 4 people	124 (38.4)	118 (47.6)
5 to 6 people	111 (34.4)	63 (25.4)
7 and more people	33 (10.2)	23 (9.3)

Household monthly income, *n* (%)		
MYR 2,500 and below	48 (18.1)	52 (24.2)
MYR 2,501 to MYR 5,000	104 (39.1)	84 (39.1)
MYR 5,001 to MYR 10,000	93 (34.9)	58 (26.9)
MYR 10,001 and above	21 (7.9)	21 (9.8)

**Table 2:** EQ-5D-5L frequencies, proportion by dimensions, level and EQ-VAS score among diabetes and cancer patients (*N* = 571)

Level/dimensions	Diabetes (*n* = 323)				

	Mobility *n* (%)	Self-care *n* (%)	Usual activities *n* (%)	Pain/discomfort *n* (%)	Anxiety/depression *n* (%)
Level 1 (no problems)	182 (56.4)	248 (76.8)	192 (59.4)	131 (40.5)	174 (53.9)
Level 2 (slight problems)	88 (27.2)	47 (14.5)	84 (26)	143 (44.3)	113 (35)
Level 3 (moderate problems)	31 (9.6)	16 (5)	32 (10)	37 (11.4)	26 (8)
Level 4 (severe problems)	14 (4.3)	5 (31.5)	10 (3.1)	9 (2.8)	7 (2.1)
Level 5 (extreme problems/unable to do)	8 (2.5)	7 (2.2)	5 (1.5)	3 (1)	3 (1)
Mean (SD)	1.693 (0.982)	1.377 (0.830)	1.613 (0.899)	1.792 (0.821)	1.613 (0.801)

EQ-VAS score					
Mean score (SD)	71.6 (18.7)				
Median (IQR)	75 (35)				

**Level/dimensions**	**Cancer patients (** ***n*** ** = 248)**				

	**Mobility** ***n*** ** (%)**	**Self-care** ***n*** ** (%)**	**Usual activities** ***n*** ** (%)**	**Pain/discomfort** ***n*** ** (%)**	**Anxiety/depression** ***n*** ** (%)**

Level 1 (no problems)	119 (47.9)	144 (58)	85 (34.3)	33 (13.3)	57(23)
Level 2 (slight problems)	67 (27)	61 (24.6)	85 (34.3)	98 (39.5)	95 (38.3)
Level 3 (moderate problems)	44 (18)	22 (8.9)	43 (17.3)	78 (31.5)	59 (23.8)
Level 4 (severe problems)	12 (4.8)	15 (6)	26 (10.5)	27 (10.9)	26 (10.5)
Level 5 (extreme problems/unable to do)	6 (2.4)	6 (2.4)	9 (3.6)	12 (4.8)	11 (4.4)
Mean (SD)	1.866 (1.027)	1.701 (1.021)	2.149 (1.115)	2.544 (1.013)	2.350 (1.080)

EQ-VAS score					
Mean score (SD)	63.7 (19.2)				
Median (IQR)	60 (98)				

**Table 3:** Mann–Whitney U test

Cost category	Diabetes rank sum (*n* = 323)	Cancer rank sum (*n* = 248)	*Z*-value *P*-value	Median (IQR)	Effect size (*r*) U statistic	Interpretation
Outpatient	84,775.5	78,530.5	−3.9500 0.0001	4.41 (3.40)	0.165 32,449.500	Small effect
Hospital	77,959.0	85,347.0	−8.3590 0.0000	5.70 (2.71)	0.350 25,633.000	Medium effect
Laboratory	85,883.0	77,423.0	−3.8350 0.0001	4.61 (1.79)	0.160 33,557.000	Small effect
Diagnostic imaging	74,372.0	88,934.0	−11.1650 0.0000	4.61 (2.08)	0.467 22,046.000	Large effect
Transportation	81,663.5	81,642.5	−5.5960 0.0000	3.91 (1.39)	0.234 29,337.500	Small to medium effect

**Table 4:** Estimated out-of-pocket expenditure among diabetes and cancer patients (*N* = 571)

Cost category	Diabetes patient (*n* = 323)			Cancer patient (*n* = 248)		

	Estimated cost (MYR)	Standard error	Visit frequency (M, SD)	Estimated cost (MYR)	Standard error	Visit frequency (M, SD)
Outpatient	116.75	17.81	2.1 (1.9)	4,922.83	2,249.08	5.6 (10.9)
Hospitalisation	1,110.01	604.98	0.7 (1.8)	12,369.69	6,236.09	2.0 (3.4)
Laboratory	136.83	44.77	1.0 (2.3)	1,266.11	528.57	2.1 (3.8)
Diagnostic imaging	828.61	829.33	0.7 (8.4)	3,383.67	1,495.04	1.9 (2.8)
Transportation	60.45	8.39	3.8 (20.8)	144.23	35.17	5.4 (10.7)

Total per 3 months	MYR 2,252.65				MYR 22,086.53	
Total per year (3 months × 4)	MYR 9,010.60				MYR 88,346.12	
